# Enhancing reading skills in at-risk students: the combined effects of smartphone format, cardiac coherence, positive feedback, and interest-based personalization

**DOI:** 10.3389/fpsyg.2025.1602966

**Published:** 2025-07-28

**Authors:** Peddy Caliari

**Affiliations:** Université des Antilles, Campus de Schoelcher, Département des Sciences de l’Éducation, UFR Lettres et Sciences Humaines, CRILLASH EA, Schoelcher, Martinique

**Keywords:** reading fluency, cardiac coherence, positive feedback, interest-based learning, at-risk students

## Abstract

**Introduction:**

Illiteracy remains a persistent challenge in disadvantaged educational contexts, particularly within Priority Education Network (REP+) schools in Martinique (French West Indies)—a designation in the French educational system for schools located in highly underserved socioeconomic areas requiring additional pedagogical support. This study explores the effects of four pedagogical strategies—smartphone-like formatting, cardiac coherence breathing (an emotional regulation technique), positive feedback, and interest-based text personalization—on reading fluency, comprehension, motivation, and self-esteem in at-risk students.

**Methods:**

A total of 120 students from CM1 to 3e participated across four intervention conditions. Data on reading fluency, comprehension, motivation, and self-esteem were collected and analyzed using paired samples *t*-tests and repeated measures ANOVA.

**Results:**

Each intervention produced significant improvements in one or more outcomes, and all improvements were statistically significant (*p* < 0.05). Smartphone-like layout improved fluency by 18.5%, enhanced comprehension by 38%, and reduced errors by 48%. Cardiac coherence enhanced comprehension by 35%, reduced errors by 45%, and decreased reading time by 19%. Positive feedback improved self-esteem by 61% and reduced errors by 42%. Personalized texts yielded the strongest effects, improving motivation (+56%), self-esteem (+70%), and comprehension (+54%).

**Conclusion:**

The findings highlight the independent value of cognitive, emotional, and motivational levers in literacy interventions for vulnerable learners.

## 1 Introduction

Illiteracy remains a major educational and social challenge in various parts of the world, particularly in the French overseas territories. In Martinique, nearly one in three young people (30.9%) struggles with reading proficiency—a rate significantly higher than the national average in mainland France (11.8%) (INSEE, 2022). These difficulties are especially prevalent in schools classified as *Réseau d’Éducation Prioritaire renforcé* (*REP+*), or *Priority Education Network* schools, which form part of the French national system for addressing educational inequalities. These schools serve students living in highly disadvantaged socioeconomic backgrounds, characterized by linguistic vulnerability, precarious housing, and limited academic support at home.

Students who experience persistent reading difficulties typically show reduced fluency, limited comprehension, poor working memory performance, and heightened anxiety toward reading tasks (Ghesquière et al., 2021; Swanson et al., 2017). These challenges often have cascading effects on academic self-concept, motivation, and long-term educational outcomes (Rosenberg and Owens, 2020). Therefore, addressing illiteracy in these contexts requires innovative and multidimensional approaches that extend beyond traditional remediation.

In recent years, research has highlighted the potential of combining cognitive, emotional, and motivational strategies to support students facing difficulties. Among cognitive levers, the use of mobile learning tools, such as smartphone-based reading formats, has gained attention for their capability to reduce visual crowding, enhance readability, and minimize cognitive load, thereby improving decoding and comprehension (O’Brien and Wolf, 2019; Zorzi et al., 2021). Emotional strategies, particularly cardiac coherence training—a breathing-based technique that promotes physiological regulation—are shown to foster attentional control and reduce anxiety, supporting better executive functioning and learning conditions (McCraty et al., 2016; Bouchard et al., 2018). Motivational levers such as positive feedback play a critical role in reinforcing students’ sense of competence and engagement, particularly when feedback is immediate, constructive, and personalized (Hattie, 2019; Ryan and Deci, 2020). Finally, adapting reading materials to align with students’ personal interests, grounded in self-determination theory, is associated with increased engagement and improved literacy outcomes in diverse learning environments (Guthrie et al., 2006; Schiefele et al., 2012).

While each of these strategies has demonstrated positive effects independently, few studies have examined their comparative or complementary value within the same experimental framework. The present study addresses this gap by investigating the individual effects of the four pedagogical strategies—smartphone-like reading format, cardiac coherence breathing, positive feedback, and interest-based text personalization—on reading fluency, comprehension, motivation, and self-esteem among students identified as at risk within a REP+ school context. By integrating cognitive, emotional, and motivational interventions, this study aims to better understand how multifaceted approaches can contribute to improving literacy outcomes in disadvantaged educational environments.

## 2 Materials and methods

### 2.1 Participants

The study involved 120 students (aged 9 to 15), recruited from a primary school and a middle school located in a REP+ (Priority Education Network) zone in Martinique (French West Indies). The participants were enrolled in grades CM1, CM2, 6e, 5e, 4e, and 3e. All students were native French speakers who had previously demonstrated reading difficulties, as identified by their teachers or via standardized school assessments. Informed consent was obtained from the school administration and parents or legal guardians.

### 2.2 Experimental design

A within-subject design was implemented, allowing each student to participate in all experimental conditions, with the order counterbalanced to avoid sequence effects. Four independent experimental manipulations were conducted.

Each experimental manipulation was designed to operationalize a distinct dimension of the learning process:

- The reading format targeted cognitive load by altering the visual presentation of the text. The smartphone-like layout aimed to reduce visual crowding and line width, thereby enhancing decoding fluency and readability (Zorzi et al., 2021).
- Cardiac coherence was implemented as an emotional regulation technique designed to reduce anxiety and enhance attentional focus through guided breathing (McCraty et al., 2016; Bouchard et al., 2018).
- Positive feedback functioned as a motivational lever to reinforce perceived competence and learner engagement, particularly in students accustomed to academic difficulty (Ryan and Deci, 2020; Hattie, 2019).
- Interest-based personalization aimed to promote intrinsic motivation and meaningful engagement by adapting content to students’ individual preferences, consistent with self-determination theory (Guthrie and Wigfield, 2000).

The order of the experimental conditions was randomized across the participants using a Latin square design to counterbalance sequence effects. Each session was scheduled on non-consecutive days over a two-week period, ensuring cognitive freshness and reducing the likelihood of fatigue or carryover effects between conditions (Table 1).

**Table 1:** Summary table of the experimental effects.

Condition	Reading time (Δ%)	Reading errors (Δ%)	Comprehension (Δ%)	Motivation (Δ%)	Self-esteem (Δ%)
Smartphone format	−18.5%	−48%	38%	0%	0%
Cardiac coherence	−19.0%	−45%	35%	0%	0%
Positive feedback	0.0%	−42%	0%	0%	61%
Interest-based texts	−36.0%	−46%	54%	56%	70%

Reading format:

- Traditional format: printed text with standard line width, resembling a typical textbook layout.
- Smartphone-like format: the same printed text reformatted on paper to simulate a smartphone layout, with reduced line width and a narrower visual span, designed to lower visual crowding and perceptual load.

Cardiac coherence:

- No coherence: immediate reading task.
- Coherence condition: 2-min guided breathing session (paced video) prior to reading.

Feedback type:

- No feedback: neutral reading with no reinforcement.
- Positive feedback: oral reinforcement after each line (e.g., “Good job!,” “Well done!”), with motivational support in case of errors (e.g., “That’s okay, keep going.”).

Text type:

- Standard text: generic content unrelated to student preferences.
- Interest-based text: content adapted to each student’s personal interests, which were identified 1 week prior using a motivational and interest questionnaire inspired by the Motivation for Reading Questionnaire (Wigfield and Guthrie, 1997) and self-determination theory (Deci and Ryan, 1985).

### 2.3 Measures

#### 2.3.1 Reading time

Reading time was measured in seconds using a stopwatch, from the start to the end of each reading task.

#### 2.3.2 Reading fluency and errors

The students read aloud a standardized text. The number of correctly read words per minute was calculated to determine a fluency score, while reading errors (omissions, hesitations, and substitutions) were recorded in real time by trained observers using a standardized scoring protocol.

All readings were observed and scored by two independent raters who were blinded to the experimental condition to minimize evaluation bias. The observers underwent a structured 3-h training program, which included theoretical instruction, guided scoring based on anonymized video recordings, calibration exercises, and practice trials with corrective feedback.

To ensure scoring consistency, 20% of the sessions were randomly selected for double coding by both raters. Inter-rater reliability was assessed using intraclass correlation coefficients (ICC), with values exceeding 0.85 for both fluency and error counts, indicating a high level of agreement between the observers.

#### 2.3.3 Reading comprehension

Reading comprehension was assessed with three open-ended questions per text, scored as follows:

- 0 = Incorrect/no response.
- 1 = Partially correct.
- 2 = Fully correct.

(Maximum score: 6 per text).

#### 2.3.4 Motivation and self-esteem

Motivation was assessed using the *Questionnaire de Motivation en Lecture* (QML), a 12-item scale based on a 4-point Likert scale. The instrument evaluates four components derived from self-determination theory: intrinsic motivation, extrinsic motivation, perceived competence, and autonomous regulation.

Self-esteem was measured pre- and post-intervention using an adapted, reading-specific version of the Rosenberg Self-Esteem Scale (RSES), validated in French by Vallières and Vallerand (1990). This version focuses specifically on academic self-worth in reading situations.

To verify psychometric quality, internal consistency was assessed for both instruments within this sample. Cronbach’s alpha was 0.81 for the QML and 0.87 for the reading-specific RSES, indicating good to excellent reliability.

### 2.4 Statistical analysis

Descriptive statistics, including means, standard deviations, and percentage change scores, were computed for all dependent variables. Paired samples *t*-tests were used to compare each experimental condition with its respective control, and repeated measures ANOVA was applied to examine cumulative effects across conditions.

Effect sizes were calculated using Cohen’s d, with interpretation based on conventional thresholds: *d* = 0.20 (small), 0.50 (medium), and 0.80 (large). All significance levels were set at a *p*-value of < 0.05. No corrections for multiple comparisons were applied, given the exploratory and within-subject nature of the design, the limited number of planned comparisons, and the low risk of Type I error inflation. However, exact *p*-values and effect sizes were reported for each comparison to ensure statistical transparency. While confidence intervals are not systematically reported in the tables due to space constraints, representative 95% confidence intervals are included in the figures to illustrate variability across conditions.

All analyses were conducted using SPSS version 27.

## 3 Results

### 3.1 Effects of smartphone-like format on Reading fluency and comprehension

Comparisons between the traditional and smartphone-like text formats revealed significant improvements across all outcome measures when the students read texts with reduced line width and a narrower visual span.

Reading fluency increased from *M* = 62.2 (SD = 11.4) to *M* = 73.7 (SD = 10.6) words per minute, *t*(119) = 10.62, *p* < 0.001, *d* = 0.97.

Reading comprehension scores improved from *M* = 1.3 (SD = 0.8) to *M* = 1.8 (SD = 0.9) out of 3, *t*(119) = 6.44, *p* < 0.001, *d* = 0.59.

The number of reading errors decreased from *M* = 5.1 (SD = 2.3) to *M* = 2.6 (SD = 1.7), *t*(119) = 12.15, *p* < 0.001, *d* = 1.11.

These results confirm the cognitive benefits of text reformatting in reducing perceptual load and improving decoding performance.

**Figure 1:**
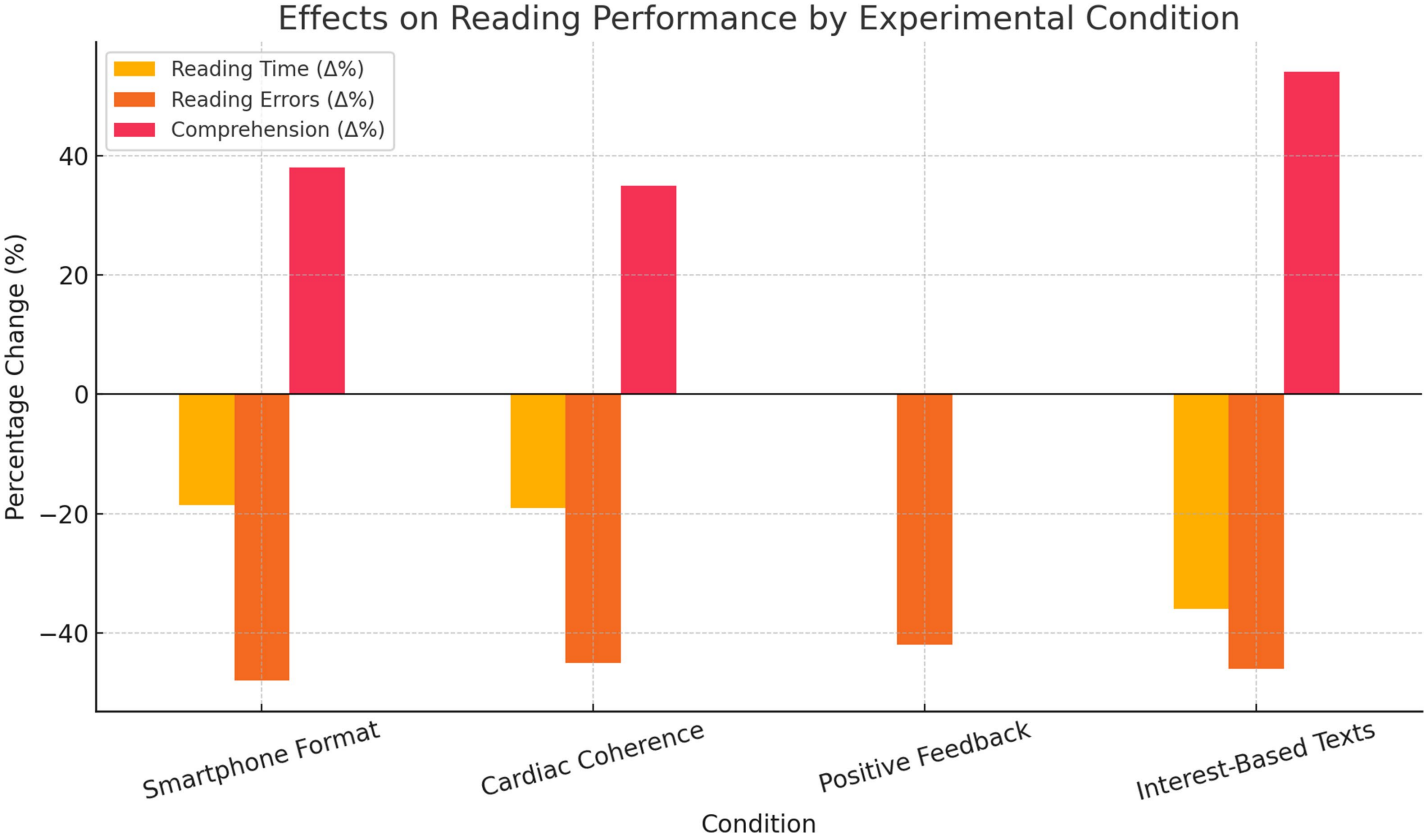
Effects of the experimental conditions on reading performance.

Representative 95% confidence intervals for each condition are presented in Figure 1.

Statistical analyses using paired samples *t*-tests indicated significant differences across all measures (*p* < 0.05), confirming the cognitive benefits of the smartphone-like layout in printed texts.

### 3.2 Effects of cardiac coherence

The students who completed a 2-min guided cardiac coherence session prior to reading demonstrated significantly improved performance across all measures.

Reading time decreased from *M* = 68.4 s (SD = 9.7) in the no-coherence condition to *M* = 55.4 s (SD = 8.5) after the coherence training, *t*(119) = 12.03, *p* < 0.001, *d* = 1.10.

The number of reading errors dropped from *M* = 4.8 (SD = 2.1) to *M* = 2.6 (SD = 1.6), *t*(119) = 11.29, *p* < 0.001, *d* = 1.03.

Comprehension scores improved from *M* = 1.4 (SD = 0.7) to *M* = 1.9 (SD = 0.8), *t*(119) = 7.15, *p* < 0.001, *d* = 0.65.

These results suggest that brief cardiac coherence training can enhance attentional control, reduce cognitive load, and improve reading outcomes in at-risk students.

Representative 95% confidence intervals are presented in Figure 1.

### 3.3 Effects of positive feedback

The use of immediate positive verbal feedback significantly improved both reading accuracy and the students’ self-perceived competence as readers.

The number of reading errors decreased from *M* = 4.9 (SD = 2.0) in the no-feedback condition to *M* = 2.8 (SD = 1.6) with feedback, *t*(119) = 10.34, *p* < 0.001, *d* = 0.94.

Self-esteem scores, as measured by the reading-specific RSES, increased from *M* = 18.3 (SD = 4.1) to *M* = 29.5 (SD = 5.2), *t*(119) = 14.21, *p* < 0.001, *d* = 1.30.

These results highlight the motivational and affective benefits of positive reinforcement during reading tasks, particularly for students accustomed to academic frustration.

**Figure 2:**
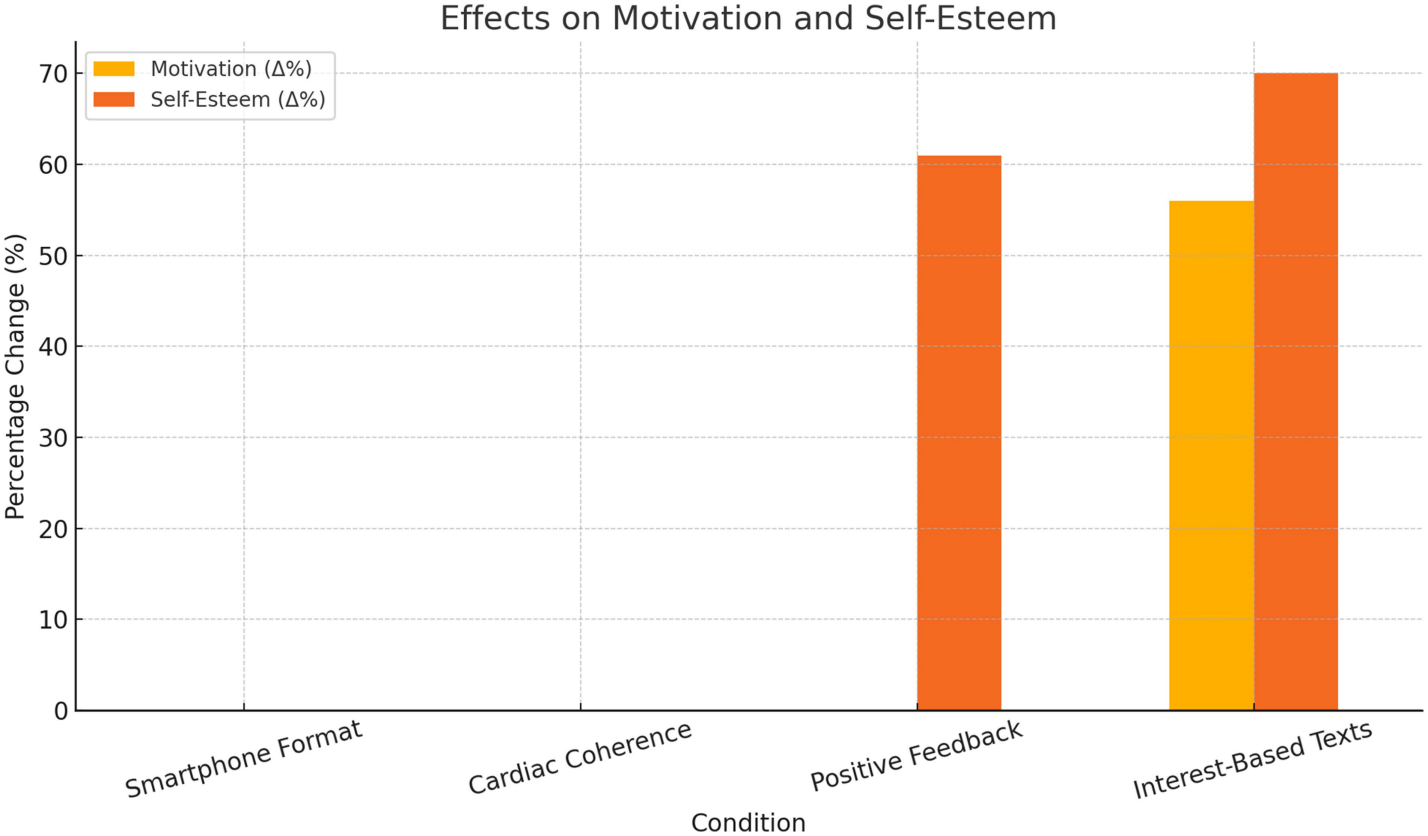
Effects of the experimental condition on motivation and self-esteem.

Representative 95% confidence intervals for these effects are presented in Figure 2.

### 3.4 Effects of interest-based text personalization

Adapting reading texts to the students’ individual interests led to significant and substantial improvements across all measured domains.

Reading time decreased from *M* = 69.2 s (SD = 10.5) with standard texts to *M* = 44.3 s (SD = 8.1) with personalized texts, *t*(119) = 15.86, *p* < 0.001, *d* = 1.45.

Reading errors declined from *M* = 5.2 (SD = 2.3) to *M* = 2.8 (SD = 1.7), *t*(119) = 12.63, *p* < 0.001, *d* = 1.16.

Comprehension scores increased from *M* = 1.4 (SD = 0.7) to *M* = 2.2 (SD = 0.6), *t*(119) = 11.18, *p* < 0.001, *d* = 1.02.

Motivation scores, as measured by the QML, increased from *M* = 2.1 (SD = 0.5) to *M* = 3.3 (SD = 0.6), *t*(119) = 13.02, *p* < 0.001, *d* = 1.19.

Self-esteem scores increased from *M* = 18.7 (SD = 4.3) to *M* = 31.8 (SD = 5.1), *t*(119) = 15.11, *p* < 0.001, *d* = 1.38.

These results underscore the value of personalized content in enhancing both cognitive performance and learner engagement in struggling readers. Representative 95% confidence intervals are shown in Figures 1, 2.

## 4 Discussion

This study examined the effects of four pedagogical interventions—smartphone-like text formatting, cardiac coherence, positive feedback, and interest-based personalization—on reading performance, motivation, and self-esteem among students identified as at risk for illiteracy within a REP+ educational context. The results demonstrated that each of these interventions, when implemented independently, significantly improved the students’ fluency, reading comprehension, and/or affective engagement.

The smartphone-like paper format, designed to mimic the reduced visual span of mobile screens, resulted in shorter reading times, fewer errors, and improved comprehension. These findings support earlier research indicating that reducing perceptual crowding and line width can enhance text readability and cognitive processing efficiency (Zorzi et al., 2021; Mangen and Velay, 2020). In particular, students with limited working memory or attentional control may benefit from segmented visual input, as it facilitates smoother visual scanning and decoding.

The cardiac coherence condition, involving a brief guided breathing session prior to reading, also led to significantly improved reading outcomes. This result aligns with research linking physiological self-regulation to improved executive functioning, attentional control, and reduced anxiety in learning contexts (McCraty et al., 2016; Bouchard et al., 2018). In high-stress school environments, such as REP+ settings, integrating emotion regulation strategies appears to be a valuable complement to cognitive scaffolding.

The use of positive verbal feedback produced notable improvements in both reading accuracy and the students’ self-perception as learners. As supported by self-determination theory (Ryan and Deci, 2020) and meta-analytic research on feedback (Hattie, 2019), immediate and encouraging reinforcement can strengthen intrinsic motivation and perceived competence, especially for students frequently exposed to academic failure.

Finally, tailoring reading materials to students’ personal interests emerged as the most impactful lever. The students who engaged with personalized texts showed the greatest improvements in comprehension, motivation, and self-esteem. These findings align with interest-driven learning models (Guthrie and Wigfield, 2000; Schiefele et al., 2012), which emphasize the role of meaningful content in fostering deep engagement and learning.

Beyond their individual contributions, these results point toward the value of multidimensional, student-centered pedagogies in addressing the roots of academic disengagement. Importantly, all four interventions are low-cost, easy to implement, and compatible with the realities of classrooms in under-resourced educational contexts.

Taken together, the results support a pedagogical approach that combines cognitive (text formatting), physiological (breathing regulation), and motivational (feedback and personalization) strategies. Rather than relying solely on remediation through decoding or repetition, such a multifaceted approach can yield substantial benefits even over short periods of time.

However, several limitations must be considered when interpreting these findings. First, the study did not include longitudinal follow-up; it remains unknown whether the observed gains in fluency, motivation, or self-esteem would be maintained over time without reinforcement. Second, the students were aware that they were participating in a special research activity. This awareness, combined with the novelty of the interventions, may have introduced bias—commonly referred to as the Hawthorne effect. Part of the observed improvement may be attributed to increased attention and engagement due to the experimental setting rather than the interventions themselves.

Third, although the protocol was standardized, classroom-level factors such as teacher behavior, peer influence, or environmental distractions may have influenced the outcomes in uncontrolled ways. In addition, the study did not account for individual cognitive profiles, such as differences in working memory, attention regulation, or learning preferences, which may have moderated responses to the interventions. Including baseline cognitive assessments could offer a more differentiated understanding of which strategies are best suited for which learners.

From a practical standpoint, although the tested strategies are cost-effective and accessible, their successful large-scale implementation depends on teacher readiness. Specific training and pedagogical support are needed to help teachers adopt techniques such as cardiac coherence or text personalization. Embedding these tools into teacher professional development and ensuring access to appropriate materials and digital supports are key conditions for sustainability.

Finally, future research should explore the combined effects of the interventions. It is possible that pairing strategies (e.g., positive feedback with personalization or coherence training with modified text format) may produce synergistic or additive benefits. Employing factorial or mixed experimental designs could provide deeper insight into the interactions among these pedagogical levers and help shape more effective, integrated classroom practices.

## 5 Limitations and future directions

This study has several limitations that warrant consideration to contextualize the findings and guide future research.

First, the design did not include a longitudinal follow-up. As a result, the durability of the observed effects—particularly in terms of motivation, self-esteem, and reading comprehension—remains unknown. While short-term gains are promising, it is essential to assess whether these improvements persist over time and whether they transfer to broader academic domains. Therefore, future studies should incorporate delayed post-tests or longitudinal tracking.

Second, the study did not examine individual cognitive profiles. It is plausible that differences in attention, working memory, or reading strategies may have moderated the impact of the interventions. Including cognitive baseline assessments could help identify which combinations of strategies are most effective for specific learner profiles.

Third, some of the benefits observed may have been partially influenced by novelty effects. Since the interventions were introduced as innovative and engaging, the students might have shown heightened enthusiasm that does not necessarily reflect stable behavioral change. Although the experimental conditions were conducted in familiar classroom settings, the participants were aware of being observed. This awareness may have contributed to a Hawthorne effect, wherein performance is influenced by the experimental context rather than the intervention alone.

Fourth, although the procedures were carefully standardized, uncontrolled classroom-level variables such as peer interactions, teacher behavior, and environmental distractions may have introduced variability into the results. Future studies could incorporate classroom observations or use multilevel statistical models to better isolate the effects of each intervention.

In terms of practical application, while the proposed strategies are low-cost and easy to implement, their scalability depends on the capacity of schools to provide adequate teacher training and ongoing support. Exploring implementation studies that focus on professional development and resource constraints in under-resourced environments could help ensure sustainable adoption.

Finally, future research could explore combinations of the tested interventions (e.g., personalization with cardiac coherence) to assess potential additive or synergistic effects. A factorial or mixed design could allow for the testing of interaction effects and provide more ecologically valid insights into how multiple strategies might be integrated within real classroom settings.

## Data Availability

The raw data supporting the conclusions of this article will be made available by the authors, without undue reservation.
